# Real-World Treatment Patterns and Survival Outcome in Advanced Anaplastic Lymphoma Kinase (ALK) Rearranged Non-Small-Cell Lung Cancer Patients

**DOI:** 10.3389/fonc.2020.01299

**Published:** 2020-08-21

**Authors:** Christian Britschgi, Alfredo Addeo, Markus Rechsteiner, Raphaël Delaloye, Martin Früh, Giulio Metro, Marco Banini, Oliver Gautschi, Sacha I. Rothschild, Peter J. Wild, Giuseppe L. Banna, Alessandra Curioni-Fontecedro

**Affiliations:** ^1^Department of Medical Oncology and Hematology, University Hospital Zurich, University of Zurich, Zurich, Switzerland; ^2^Geneva University Hospitals (HUG), Geneva, Switzerland; ^3^Institute of Pathology and Molecular Pathology, University Hospital Zurich, Zurich, Switzerland; ^4^Department of Oncology and Hematology, Cantonal Hospital St. Gallen, St. Gallen, Switzerland; ^5^Department of Oncology, University Hospital of Bern, Bern, Switzerland; ^6^Medical Oncology, Azienda Ospedaliera Di Perugia, Perugia, Italy; ^7^University of Bern and Cantonal Hospital of Lucerne, Lucerne, Switzerland; ^8^Department of Internal Medicine, Medical Oncology, University Hospital Basel, Basel, Switzerland; ^9^Portsmouth Hospitals NHS Trust, Portsmouth, United Kingdom

**Keywords:** real world data, NSCLC, targeted therapy, overall survival, ALK-rearranged non-small cell lung cancer

## Abstract

**Introduction:** Survival of ALK-rearranged NSCLC patients has dramatically improved by the use of multiple ALK-tyrosine kinase inhibitors (ALK-TKI). However, still little is known about the impact of drug sequencing and clinical features on survival in a real-world setting.

**Methods:** Patients with stage IV ALK-rearranged NSCLC treated at six centers in Switzerland and Italy were identified and standard clinical variables collected. OS curves were constructed using the Kaplan–Meier method and compared with the log-rank test. Multivariate Cox proportional hazard analysis was applied to determine the correlations between clinical features and OS. In four patients, biopsies were subjected to NGS.

**Results:** One-hundred and twenty-one patients with stage IV ALK-rearranged NSCLC diagnosed between 2011 and 2016 were included. With a median follow-up time of 39.5 months, the median OS from diagnosis of stage IV disease was 48.0 months. First-line treatment consisted of an ALK-TKI in 24% of patients, with crizotinib in 83% of them. Chemotherapy as first-line treatment did not influence OS (*p* = 0.955). The use of more than one ALK-TKI line positively correlated with OS (*p* = 0.016), as well as the use of alectinib or lorlatinib in any treatment line, as compared to the use of crizotinib ± ceritinib (*p* = 0.022). A never smoking history was an independent prognostic factor for OS (*p* = 0.032). Moreover, treatment with alectinib significantly improved OS.

**Conclusions:** Targeted treatment for ALK-positive NSCLC patients lead to prolonged OS. Smoking status was a negative independent prognostic factor in a multi-variate analysis. The use of alectinib or lorlatinib in any treatment line improved overall outcome.

## Introduction

Over the past years, several key improvements in the treatment of non-small cell lung cancer (NSCLC) have been made. In particular, the identification of genetic alterations responsible for initiation and maintenance of the malignant phenotype in several cancers including NSCLC has led to the development of targeted therapies. These new molecules specifically target the so-called oncogenic driver mutation and have improved patient outcomes, in terms of overall survival (OS), but also in terms of quality of life (QoL). Among different oncogenic driver mutations known in NSCLC, rearrangements of the *ALK* (anaplastic lymphoma kinase) gene have been a particularly successful target in terms of the development of new targeted therapies, achieving important improvements in survival (1–6). *ALK* gene rearrangements are present in about 5% of NSCLC patients (7). They were first identified in a resected adenocarcinoma specimen from a 62-year-old male smoker, and can involve different fusion partners presenting as either inversions or translocations (8, 9). Inversions on the short arm of chromosome 2, which juxtapose echinoderm microtubule-associated protein-like 4 (*EML4*) with *ALK* and produce EML4-ALK-fusion proteins are the most commonly observed rearrangement, but at least 27 fusion variants have been identified so far (2, 10).

Identification of an *ALK* rearrangement is of therapeutic relevance at the time of initiation of first-line treatment, as it confers sensitivity to ALK tyrosine kinase inhibitors (ALK-TKIs). Importantly, the rare occurrence of KRAS co-mutations leads to primary resistance to ALK-TKIs (11). These targeted therapies have resulted in major clinical advances over the last decade, with superior objective response rates (ORRs) and progression-free survival (PFS) compared to conventional chemotherapy in *ALK* rearranged patients, initially shown for the first generation ALK-TKI crizotinib (4, 12). Despite the initial efficacy of crizotinib, patients progress after a median time of about 12 months, with the brain being the most frequent site of progression or relapse (13). This is due to the development of resistance mechanisms, the most frequent one is the acquisition of point mutations in the rearranged *ALK* gene. Classically, second-generation ALK inhibitors such as alectinib or ceritinib have emerged as standard of care in crizotinib-resistant patients (14–17). Later, third-generation inhibitors such as lorlatinib or the second-generation inhibitor brigatinib were shown to overcome resistance to first and even second-generation ALK-TKIs (18–21). However, each ALK-TKI has a different spectrum of sensitivity to ALK-resistance mutations (22). In the light of these findings, identifying the various resistance mechanisms is becoming more important to select further treatments and overcome acquired resistance (2).

Here, we report the results of the transalpine ALK registry, a collaborative real-world study performed in five institutions in Switzerland and one in Italy. The goal was to collect outcome data of patients with ALK-rearranged NSCLC, with a particular focus on overall survival (OS) by the use of ALK-TKIs and their sequencing.

All the clinical ALK-rearranged NSCLC patients' data were retrospectively collected and analyzed. If available, biopsies both before starting ALK-TKI and at progression were subjected to next-generation sequencing (NGS) to further explore resistance mechanisms. All this study was approved by the local ethics committee (EK-ZH-2018-01919) and in accordance with the local laws and regulations.

## Materials and Methods

### Study Design

This is a retrospective study study performed in collaboration among Swiss and Italian cancer centers. Data on patients with metastatic *ALK*-rearranged NSCLC on treatment with an ALK-TKI were collected by each participating center and assembled in a central database.

### Patients Characteristics

Several demographic and background clinical characteristics were documented for each patient, such as age, gender, smoking status, histology, patterns of metastases, co-mutations, and treatments received in any line. Since the ALK-TKIs were utilized in different lines of systemic treatment, the OS was calculated from the date of diagnosis to the date of death or last time the patient was seen. Patients still alive at the time of data collection were censored at the date of the last available medical record. Several other clinical endpoints were also assessed: objective response rate (ORR) was defined as the proportion of patients achieving either a complete or a partial remission as best clinical response to an ALK-TKI according to the local radiologists' interpretation based on the response Evaluation Criteria for Solid Tumors (RECIST) 1.1 or clinically, when indicated. The ORR rate, as well as the progression-free survival, were not reported for the overall population due to the different treatment lines, in which ALK-TKI were administered.

The inclusion criteria were as follows: diagnosed with metastatic NSCLC, confirmation of an *ALK* gene rearrangement by standard diagnostic procedures used in the respective institution, as recorded in the patient's medical record, 18 years of age or older at diagnosis of *ALK*-rearranged NSCLC. If a biopsy at diagnosis and at progression was available, those samples were subjected to NGS.

### Statistical Analyses

Descriptive statistics were used to summarize patients' demographic and treatment characteristics at diagnosis and subsequent recurrence or progression. All analyses were conducted using SigmaPlot statistical software (Version 12.5; San Jose, CA, USA). OS was analyzed using the Kaplan–Meier method and was reported as median with confidence limits (95% confidence intervals, CI), with statistical significance of survival differences assessed using a non-parametric log-rank test. In order to study any possible influence of main prognostic factors on the OS, multivariate analysis was performed according to the Cox proportional hazards survival model considering statistically significant *p* <0.05. All statistical analyses were performed using two-sided tests. All data were analyzed for the pooled study sample comprising patients from Italy and Switzerland combined.

### Molecular Analysis

In four patients, pre- and post-treatment biopsies were subjected to NGS using the Ion AmpliSeq Comprehensive Cancer Panel (CCP), or the Thermo Fisher Oncomine Focus Assay Panel (OFA). NGS was performed centrally at one of the sites (University Hospital Zurich). The variant calling was done using Ion Reporter (Thermo Fisher Scientific). Only variants predicted to be damaging by SIFT and PolyPhen were included. Additionally, the variant frequency cut-off was adjusted to the estimated tumor cell content in each specimen.

## Results

### Patient Characteristics

Between January 2011 and June 2016, a total of 121 patients at six centers were identified. Break-apart fluorescence *in situ* hybridization was used in almost all cases to detect *ALK* rearrangement (*n* = 119). In the remaining two cases, ALK immunohistochemistry was performed. Baseline patients' characteristics are summarized in Table 1. Fifty-six were male and 65 female, median age was 52 years old (range 19–81). Histology was adenocarcinoma in 111 cases (92%). The majority of the patients were never smokers (56%, 58 patients, out of 104 assessable patients). Of 121 patients, 98 presented with stage IV disease (98/121; 92%), 37 (37/121; 31%) with brain metastases at the time of diagnosis; additionally, 14 patients (14/84; 17%) newly developed brain metastases under therapy. 115 received first-line treatment with either chemotherapy (ChT) (*n* = 86, 71%), or ALK-TKIs (*n* = 29, 24%). Seventy-five patients (62%) received ALK-TKIs after first-line treatment, 58 (48%) as second-line treatment. Forty-six patients (45%) received more than one ALK-TKI treatment line, including the use of alectinib or lorlatinib in 26 of them (25%). Seventeen patients (14%) have not received a treatment with ALK-TKI at the time of analysis (of these 17 patients, 6 patients started with first line chemo and were either still controlled/even in complete remission at censoring, or were converted to be treated in an oligo-metastatic approach and have never relapsed since then; eight patients have started with first line chemo and have died before ever having had the chance to get a TKI; form three patients only data on survival were available).

**Table 1:** Characteristics of patients (*n* = 121).

**Parameter**	***n* (%)**	
	**Patients receiving TKI**	**Patients without TKI**
	104	17
**Region of origin**		
Italy/Switzerland	42/62 (40/60)	1/16 (6/94)
**Gender**		
Female/Male	55/49 (53/47)	10/7 (58/42)
Age, median	52 (19–81 year)	61 (36–79)
**Tobacco**		
Never/former/current smokers	52/16/20 (50/15/19)^a^	6/8 (35/47)
Unknown	16 (15)	3 (18)
**Histology**		
Adenocarcinoma	95 (91)	16 (94)
Squamous/others	9 (8)	1 (6)
**Stage at diagnosis**		
I/II/III	3/2/15 (3/2/14)	0/2/1 (0/11/6)
IV	84 (81)	14 (82)
≥3 involved organs	46 (44)	5 (29)
BMs	33 (33)^a^	4 (23)
BMs treated with RT	24 (72)^b^	4 (100)
BMs new on treatment	13 (19)^c^	1 (8)
1st line CHT	75 (72)	11 (65)
1st line TKI	29 (24)	
Crizotinib/alectinib/ceritinib	24/3/2 (83/10/7)	
>1st line TKI	75 (62)	
2nd/3rd/4th/5th/6th/8th line	58/9/3/2/2/1 (48/7/2/2/2/1)	
One TKI line	57 (55)^d^	
>One TKI line	46 (45)^d^	
Use of alectinib/lorlatinib	26 (25)^e^	
Use of crizotinib ± ceritinib	78 (75)^e^	
No use of TKI	17 (14)	

a Of 103 assessable patients.

b Of patients with BMs.

c Of patients without BMs at diagnosis.

d Of assessable patients.

e Of 103 assessable patients.

*BMs, brain metastases; CHT, chemotherapy; CI, confidence interval; OS, overall survival; RT, radiotherapy; TKI, tyrosine kinase inhibitor*.

### Outcome

**Figure 1:**
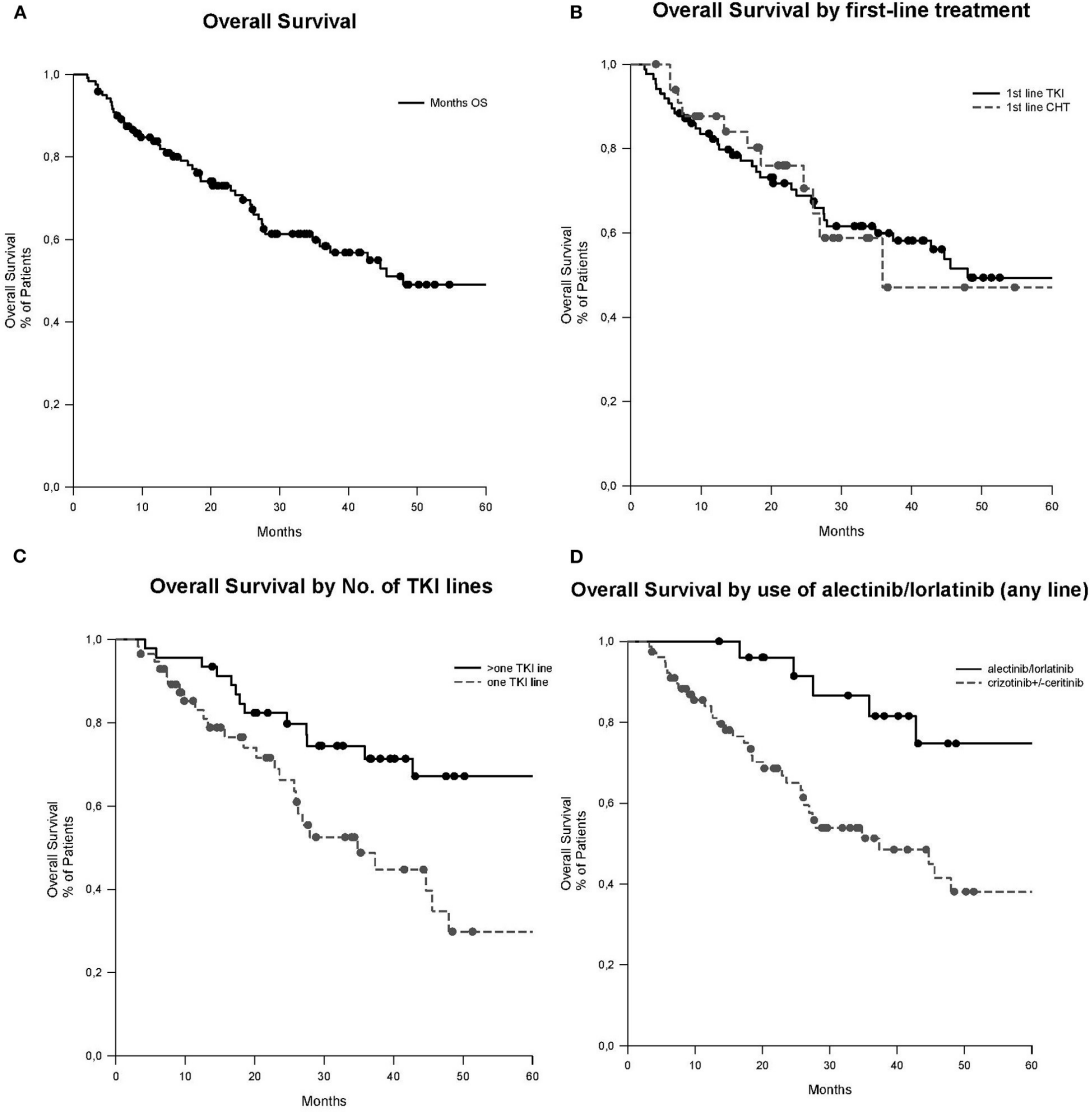
OS curves (median follow-up of 39.5 months, 95% CI: 32.1–77.7). **(A)** OS of all patients, median 48.0 months (95% CI: 12.9–83.0). **(B)** OS according to the first-line of treatment, first-line TKI median 35.8 months (95% CI NA) vs. first-line ChT 48.0 months (95% CI: 11.8–84.1) (*p* = 0.955). **(C)** OS according to the number of lines of TKIs, >one line of TKIs median 85.7 months (95% CI: 63.9–107.5) vs. one line of TKI 34.8 months (95% CI: 21.6–48.0; *p* = 0.016). **(D)** OS according to the use of alectinib or lorlatinib in any line. Alectinib/lorlatinib median 85.7 months (95% CI: 64.0–107.5) vs. crizotinib ± ceritinib 37.3 months (95% CI: 16.1–58.6; *p* = 0.022). ChT, chemotherapy; CI, confidence interval; NA, not assessable; No., number; OS, overall survival; TKI, tyrosine kinase inhibitor.

With a median follow-up of 39.5 months (95% confidence interval [CI]: 32.1–77.7), median OS was 48.0 months (95% CI: 12.9–83.0). A non-significant trend in OS was observed between patients with or without brain metastases at diagnosis (median OS 34.8 vs. 72.4 months, respectively, *p* = 0.323), with no significant difference in OS between those treated or not with brain radiotherapy (median OS not reached vs. 24.6 months, respectively, *p* = 0.567). No significant difference in OS was observed between patients treated with ALK-TKIs or ChT as first-line treatment (*p* = 0.955). A significant difference in OS was observed in favor of patients treated with more than one treatment line of ALK-TKIs as compared to those treated only with one line of ALK-TKI (median OS of 85.7 vs. 34.8 months, respectively, *p* = 0.016) and whose ALK-TKIs included alectinib or lorlatinib (median OS of 85.7 vs. 37.3 months, respectively, *p* = 0.022; see Table 2 and Figure 1). In multivariate analysis, never smoker status was the only independent prognostic factor associated with better OS (Hazard Ratio [HR] 0.499, 95% CI: 0.265–0.941, *p* = 0.032). A non-significant trend toward a better prognosis was observed for adenocarcinoma histology (HR 0.418, 95% CI: 0.175–1.002, *p* = 0.051; see Table 3).

**Table 2:** Patients' outcome (*n* = 121).

**Parameter**	**Median,** **months**	**95% CI**	**Log-rank *p*-value**
Follow-up	39.5	32.1–77.7	
OS	48.0	12.9–83.0	
OS no BMs	72.4	33.0–111.7	0.323
OS BMs	34.8	NA	
Brain RT	NR	NA	0.567^a^
No brain RT	24.6	20.2–29.0	
1st line TKI	35.8	16.9–54.7	0.526
>1st line TKI	72.4	29.3–115.4	
1st line TKI	35.8	NA	0.955
1st line CHT	48.0	11.8–84.1	
One TKI line	34.8	21.6–48,0	0.016
>One TKI line	85.7	63.9–107.5	
Use of alectinib/lorlatinib	85.7	64.0–107.5	0.022
Use of crizotinib ± ceritinib	37.3	16.1–58.6	

a On 37 patients with BM only.

*BMs, brain metastases; CHT, chemotherapy; CI, confidence interval; NA, not assessable; NR, not reached; OS, overall survival; RT, radiotherapy; TKI, tyrosine kinase inhibitor*.

**Table 3:** Cox multivariate analysis on OS.

**Parameter**	**HR**	**95% CI**	***p*-value**
Gender (female vs. male)	1.191	0.645-2.199	0.576
Tobacco (never vs. other)	0.499	0.265-0.941	0.032
Histology (adenocarcinoma vs. other)	0.418	0.175-1.002	0.051
Stage (IV vs. other)	2.187	0.860-5.563	0.100
Brain metastases (yes vs. no)	1.172	0.560-2453	0.673
Organ (3 or more vs. less)	1.653	0.795-3.437	0.178

*CI, confidence interval; HR, Hazard Ratio; OS, overall survival*.

#### Analysis of Resistance to ALK-TKIs

**Figure 2:**
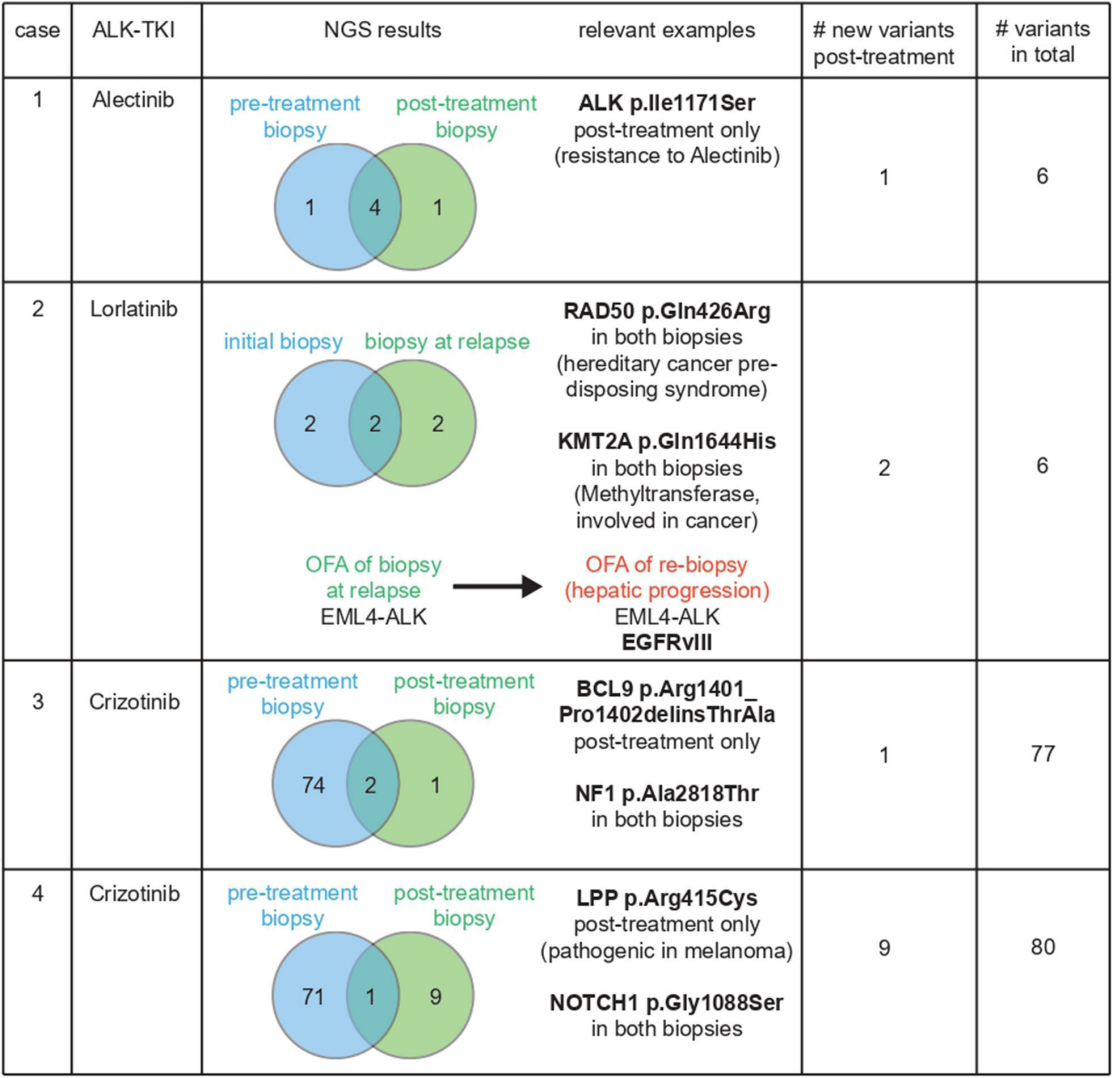
Summary of molecular aberrations in pre- and post-treatment biopsies.

Case #1 was a male with a history of cisplatin/pemetrexed as a first-line therapy, with disease stabilization for 6 months. Upon progression, he was started on crizotinib with a partial remission (PR) as best response, which lasted for 7 months. He was first oligo-progressing and received a pulmonary lobectomy of the left lower lobe with continuation of crizotinib, but was switched to docetaxel three months later upon systemic progression. After two months he was progressing and switched to ceritinib for 5 months, but was then progressing in the brain. After that, alectinib was initiated, which achieved a PR for almost 1.5 years. He then progressed in the liver and a hepatic lesion was biopsied. This biopsy and the lobectomy specimen were subjected to NGS using the CCP and an ALK p.Ile1171Ser point mutation was detected in the liver biopsy, which was absent before, most likely explaining the resistance to alectinib (Figure 2).

Case #2 was a female patient who was diagnosed with stage IIIA adenocarcinoma of the lung. She was operated upfront and received an adjuvant chemotherapy with four cycles of cisplatin/pemetrexed, but relapsed 7 months later with metastases in retroclavicular lymph nodes. At that point, an ALK-translocation was diagnosed and she was started on crizotinib. Upon progression, she received cisplatin/pemetrexed again, later a re-challenge, followed by docetaxel and then ceritinib. Upon progression in the brain and liver she was started on lorlatinib. After an initial response she was progressing in the liver and in the bones. An NGS analysis performed on a biopsy of a progressing hepatic lesion using the OFA panel at that stage showed expression of the EGFR variant III with deletion of exons 2–7 (EGFRvIII, Figure 2). This EGFR variant has been discussed to be potentially immunogenic and was assessed in clinical trials in glioblastoma (23). The patient was started on nivolumab and responded for 6 months.

Case #3 was a male patient with a stage IV (bone) adenocarcinoma of the lung. After an initial course of palliative cisplatin/pemetrexed he was started on crizotinib and was responding for 14 months. He then presented with oligo-progressive disease (oligo-PD) in two locations (bone), on which he was irradiated while continuing crizotinib. However, 2 months later he was progressing in the primary tumor, which was biopsied. The initial diagnostic specimen and this re-biopsy were subjected to NGS using the CCP, which showed emergence of a BCL9 p.Arg1401_Pro1402delinsThrAla mutation (Figure 2). The primary tumor was then irradiated and the patient continued on crizotinib for more than 1.5 years until he deceased suddenly at home.

Case #4 was a female patient with stage IV (malignant pleural effusion) adenocarcinoma of the lung. She received one cycle of palliative cisplatin/pemetrexed, but due to side effects and her reduced general condition was switched to crizotinib shortly thereafter. She was progressing 7 months later, at which timepoint a broncho-alveolar lavage (BAL) was performed. Cells from this BAL and cells from the initial malignant pleural effusion were subjected to NGS using the CCP, which showed a newly emergent LPP p.Arg415Cys mutation. The patient was then started on ceritinib with a partial remission as best response, which was maintained for 6 months. She then deteriorated rapidly and eventually died due to a pulmonary infection and pulmonary PD.

## Discussion

Patients with *ALK*-fusion positive stage IV NSCLC represent a subgroup with favorable OS compared to patients with NSCLC and no actionable molecular alterations. Real-life data allow for evaluating the impact of treatment sequences, as well as giving detailed information about clinical features, which can be associated with patients outcome. Here we present the analysis of 121 *ALK*-fusion-positive patients treated in several institutions with comprehensive data and access to ALK-TKI through early access programs and/or clinical trials with ALK-TKI. In this cohort of ALK positive patients, the median OS was 48 months. This reflects the impressive efforts made in the development and access to ALK-TKIs when compared to NSCLC patients without actionable molecular alterations. Since the very first results with crizotinib in late and first-line, the treatment of these patients has dramatically changed (2). However, OS survival from the PROFILE-1014 differs based on the access to further ALK-TKI after crizotinib, with a 5 year survival rate of 75 vs. 28% in patients who received subsequent ALK-TKI or not, respectively (4, 24). Indeed, also in our cohort of patients, the major impact on survival was given by the access to subsequent ALK-TKIs with patients receiving subsequent ALK-TKI having a median OS of 85.7 vs. 34.8 months if they did not. These data closely resemble previous observations by Pacheco et al. (25) reporting a median overall survival of 80 months, where about 80% of the patients received subsequent ALK-TKIs and 89.6 months reported by Duruisseaux in patients receiving next-generation ALK-TKIs compared to 28.2 months when they did not (26). On the other hand a median OS of 49 months was reported by Gainor, as 70% received only ceritinib as second line ALK-TKI (17). Accessibility to drugs and performance status might play a role in the patients who underwent subsequent treatment lines, which is difficult to discriminate retrospectively.

We could not evaluate the impact of alectinib given in first vs. further lines of therapy due to the small number of patients receiving it in first-line (*n* = 3). In order to identify possible clinical features with impact on patients survival, we first evaluated the localization pattern and number of metastases at time of diagnosis. Clinical characteristics of this patient cohort did not differ from the ones reported in clinical trials, e.g., brain metastases were present in 30% of patients at time of diagnosis. It has been previously reported that liver and multiple-organ metastases might have a negative prognostic impact in patients with advanced adenocarcinoma of the lung (27). In our *ALK*-rearranged NSCLC cohort, however, the number or localizations of metastases did not have any impact on survival. In line with previous reports, the presence of brain metastases in patients with *ALK*-fusion-positive NSCLC had no impact on patients outcome, and neither did radiotherapy of brain metastases. Only a positive smoking history was a significant prognostic factor for worse survival. About 45% of patients were current or former smokers, which underscores the importance of testing all patients for *ALK* gene rearrangements, irrespective of their smoking status. *ALK* testing was almost exclusively performed by FISH at time of diagnosis, and in four cases the analysis of resistance mechanisms to ALK-TKI was performed using a tissue biopsy. Molecular analysis included NGS, which allowed the detection of *ALK* mutations previously described to be associated with resistance to crizotinib and one patient with expression of an alternative splice variant of EGFR (EGFRvIII). This is the first report of such an alteration as a potential mechanism of resistance to lorlatinib. The EGFRvIII splice variant has been reported in solid tumors and has been reported to generate a highly immunogenic peptide that is currently being studied as target for immunotherapeutic approaches (23). In our patient, based on this result, a treatment with an anti PD-1 antibody (nivolumab) as monotherapy was initiated, leading to a partial response. This is of major interest as *ALK*-fusion-positive patients are usually excluded from clinical trials with immunotherapies due to reported lack of responses (28, 29). This discovery underlines the importance of detecting and understanding new mechanisms of resistance in this population of patients.

Taken together, an analysis of real-world data allowed for understanding the dynamics of tumor evolution in ALK-fusion-positive patients under specific ALK-TKIs. Understanding the resistance mechanisms that evolve during this treatment journey for each patient is key toward the general aim of precision medicine. In particular, access to further lines of ALK-TKIs had a significant impact on survival of a broad population of *ALK*-fusion-positive patients who represent every-day patients seen in clinical practice. The transalpine registry has fostered a collaborative effort between different institutions with the intent to learn more from each single case, to give access to drugs, and to search for resistance mechanisms to ALK-TKI; an issue still under debate with newly discovered implications for patients care.

## Ethics Statement

The studies involving human participants were reviewed and approved by Zurich Ethical committee, (EK-ZH-2018-091919) and all participants provided informed consent to participate in this study. Written informed consent was obtained from every patient for the publication of any potentially identifiable images or data included in this manuscript.

## Author Contributions

All authors listed have made a substantial, direct and intellectual contribution to the work, and approved it for publication.

## Conflict of Interest

CB reports personal fees from AstraZeneca, Pfizer, Takeda, Roche, Janssen-Cilag, Novartis and Boehringer-Ingelheim; non-financial support from AstraZeneca and Takeda; all outside the submitted work. AA reports personal fees from AstraZeneca, Pfizer, Takeda, Roche, Takeda, MSD, and BMS; grants and personal fees from Boehringer-Ingelheim; all outside the submitted work. RD reports personal fees from Janssen-Cilag, Boehringer-Ingelheim, and IPSEN pharma, outside the submitted work. MF reports grants and other from BMS; other from BMS, MSD, Astra Zeneca, Boehringer-Ingelheim, Roche, and Takeda; grants from Astra Zeneca; all outside the submitted work. GM reports personal fees from Boehringer-Ingelheim and non-financial support from AstraZeneca, outside the submitted work. SR reports grants, non-financial support and other from AstraZeneca, Roche, and Boehringer-Ingelheim; personal fees, non-financial support and other from Takeda; non-financial support, and other from MSD Oncology and Bristol-Myers-Squibb; other from Pfizer, Novartis, Eli Lilly, Eisai; grants and other from Meck Serono; grants from AbbVie; all outside the submitted work. PW reports personal fees from AstraZeneca, Pfizer, Takeda, Roche Janssen-Cilag, Novartis, and Boehringer-Ingelheim, outside the submitted work. GB reports personal fees from Janssen-Cilag, Boehringer Ingelheim, and Roche; non-financial support from Bristol-Myers Squibb, AstraZeneca/MedImmune, Pierre Fabre, and Ipsen; all outside the submitted work. AC-F reports personal fees from Astra Zeneca, Roche, Pfizer, Takeda, BMS, Boehringer Ingelheim, and MSD, outside the submitted work. The remaining authors declare that the research was conducted in the absence of any commercial or financial relationships that could be construed as a potential conflict of interest.
